# METTL3 and METTL14-mediated N^6^-methyladenosine modification of SREBF2-AS1 facilitates hepatocellular carcinoma progression and sorafenib resistance through DNA demethylation of SREBF2

**DOI:** 10.1038/s41598-024-55932-7

**Published:** 2024-03-14

**Authors:** Xianjian Wu, Min Zeng, Yunyu Wei, Rongzhou Lu, Zheng Huang, Lizheng Huang, Yanyan Huang, Yuan Lu, Wenchuan Li, Huamei Wei, Jian Pu

**Affiliations:** 1 Department of Hepatobiliary Surgery, Affiliated Hospital of Youjiang Medical University for Nationalities, No. 18 Zhongshan Two Road, Baise, 533000 China; 2 Graduate College of Youjiang Medical University for Nationalities, Baise, China; 3 Clinical Pathological Diagnosis and Research Center, Affiliated Hospital of Youjiang Medical University for Nationalities, Baise, China; 4 Guangxi Clinical Medical Research Center of Hepatobiliary Diseases, Baise, China

**Keywords:** Hepatocellular carcinoma, N^6^-methyladenosine, DNA methylation, Sorafenib resistance, SREBF2, Cancer epigenetics, DNA methylation, Hepatocellular carcinoma

## Abstract

As the most prevalent epitranscriptomic modification, N^6^-methyladenosine (m^6^A) shows important roles in a variety of diseases through regulating the processing, stability and translation of target RNAs. However, the potential contributions of m^6^A to RNA functions are unclear. Here, we identified a functional and prognosis-related m^6^A-modified RNA SREBF2-AS1 in hepatocellular carcinoma (HCC). The expression of SREBF2-AS1 and SREBF2 in HCC tissues and cells was measured by RT-qPCR. m^6^A modification level of SREBF2-AS1 was measured by methylated RNA immunoprecipitation assay. The roles of SREBF2-AS1 in HCC progression and sorafenib resistance were investigated by proliferation, apoptosis, migration, and cell viability assays. The regulatory mechanisms of SREBF2-AS1 on SREBF2 were investigated by Chromatin isolation by RNA purification, RNA immunoprecipitation, CUT&RUN, and bisulfite DNA sequencing assays. Our findings showed that the expression of SREBF2-AS1 was increased in HCC tissues and cells, and positively correlated with poor survival of HCC patients. m^6^A modification level of SREBF2-AS1 was also increased in HCC and positively correlated with poor prognosis of HCC patients. METTL3 and METTL14-induced m^6^A modification upregulated SREBF2-AS1 expression through increasing SREBF2-AS1 transcript stability. Functional assays showed that only m^6^A-modified, but not non-modified SREBF2-AS1 promoted HCC progression and sorafenib resistance. Mechanistic investigations revealed that m^6^A-modified SREBF2-AS1 bound and recruited m^6^A reader FXR1 and DNA 5-methylcytosine dioxygenase TET1 to *SREBF2* promoter, leading to DNA demethylation at *SREBF2* promoter and the upregulation of *SREBF2* transcription. Functional rescue assays showed that SREBF2 was the critical mediator of the oncogenic roles of SREBF2-AS1 in HCC. Together, this study showed that m^6^A-modified SREBF2-AS1 exerted oncogenic roles in HCC through inducing DNA demethylation and transcriptional activation of *SREBF2*, and suggested m^6^A-modified SREBF2-AS1 as a prognostic biomarker and therapeutic target for HCC.

## Introduction

Liver cancer is one of most lethal malignancies in the world, with the incidence rate ranking the sixth and the mortality ranking the third, according to the global cancer statistics^1^. Hepatocellular carcinoma (HCC) is the major pathological subtype, which accounts for more than 90% of liver cancer^2^. For early stages HCCs, surgical resection is the standard and most efficient therapy^3^. However, many HCC patients are diagnosed at the advanced stages and therefore are not suitable candidates for surgery^3^. For HCCs with advanced stages, molecule-targeted therapy and immunotherapy are recommended, including sorafenib, lenvatinib, bevacizumab, atezolizumab, et al^4^. However, many HCC patients are not sensitive to these therapies^5,6^. Therefore, it is urgent to elucidate the mechanisms underlying HCC progression and drug resistance.

Apart from genetic mutation, epigenetic aberrations, including DNA methylation, histone modification, and non-coding RNAs, also contribute to HCC initiation and progression through regulating the expression of oncogenes and/or tumor suppressors^7–10^. Recently, epitranscriptomic modifications are revealed to be another critical regulatory manner to control gene expression^11^. N^6^-methyladenosine (m^6^A) is the most prevalent internal modification on messenger RNAs (mRNAs) and long non-coding RNAs (lncRNAs)^12,13^. Accumulating evidences revealed that m^6^A modification is involved in various physiopathological processes^14–16^. Aberrant m^6^A modification is also frequently founded in many diseases^17–19^. m^6^A regulates diseases progression through influencing the processing, stability, and/or translation of target RNAs^20–22^. Our previous study also found that m^6^A modification level of FAM111A-DT was increased in HCC^23^. m^6^A modification increases the stability of FAM111A-DT, which exerts oncogenic roles in HCC^23^. m^6^A is catalyzed by methyltransferase complex, including METTL3, METTL14, WTAP, as well as other additional partner proteins^24–27^. m^6^A modification is reversible by demethylases, such as FTO and ALKBH5^28–30^. m^6^A is recognized and bound by m^6^A readers, such as YTH domain-containing family and IGF2BPs, which mediate the roles of m^6^A in regulating RNA fate^31,32^.

As a critical epigenetic modification manner, lncRNA shows important roles in a variety of diseases^33–35^. Several reports demonstrated that m^6^A modification regulates the processing and stability of lncRNAs^36–38^. However, whether m^6^A modification regulates the functions of lncRNAs are still largely unknown. Several studies searched the m^6^A-modified lncRNAs which were correlated with prognosis of HCCs^39–42^. Among these lncRNAs, we noted lncRNA SREBF2-AS1, which was revealed to be an m^6^A-related and prognosis-related lncRNA in HCC by two independent groups^41,42^. Here, we further investigated the expression, roles, and mechanisms of m^6^A-modified SREBF2-AS1 in HCC. We found that not only the expression level, but also m^6^A modification level of SREBF2-AS1 was correlated with prognosis of HCCs. Furthermore, we found that only m^6^A-modified SREBF2-AS1, but not non-modified SREBF2-AS1 promoted HCC progression and sorafenib resistance. Mechanistic investigations revealed that m^6^A-modified SREBF2-AS1 induced DNA demethylation at *SREBF2* promoter and upregulation of SREBF2, which mediated the roles of m^6^A-modified SREBF2-AS1 in HCC.

## Materials and methods

### Tissue samples

Eighty pairs HCC tissues and matched noncancerous liver tissues were acquired with written informed consents from HCC patients who received surgical resection at the Affiliated Hospital of Youjiang Medical University for Nationalities. This study was performed following the Declaration of Helsinki. The Affiliated Hospital of Youjiang Medical University for Nationalities Institutional Review Board reviewed and approved this study (Approval Number: YYFY-LL-2023-127). All methods were performed in accordance with the relevant guidelines and regulations.

### Cell culture and treatment

Human HCC cell lines HepG2 (cat. no. HB-8065) and SNU-398 (cat. no. CRL­2233), and human immortalized liver cell line THLE-2 (cat. no. CRL-2706) was obtained from American Type Culture Collection (ATCC, Manassas, VA, USA). Human HCC cell line HuH-7 (cat. no. SCSP-526) was acquired from the Chinese Academy of Sciences Cell Bank (Shanghai, China). HepG2, SNU-398, and HuH-7 cells were cultured in Eagle's Minimum Essential Medium (Invitrogen, Carlsbad, CA, USA), RPMI 1640 medium (Invitrogen), and Dulbecco’s modified Eagle’s medium (Invitrogen) respectively, supplemented with 10% fetal bovine serum (Invitrogen). THLE-2 was maintained using the BEGM Bullet Kit (cat. no. CC-3170, Lonza, Basel, Switzerland). Growth conditions were 37 °C, 5% CO_2_. All cells were routinely tested as mycoplasma-free.

### Quantitative polymerase chain reaction (qPCR)

Total RNA was extracted from indicated tissues and cells using the RNA isolater Total RNA Extraction Reagent (cat. no. R401, Vazyme, Nanjing, China). Reverse transcription (RT) was performed using the RNA and the HiScript III RT SuperMix for qPCR (cat. no. R323, Vazyme). qPCR was performed using the ChamQ Universal SYBR qPCR Master Mix (cat. no. Q711, Vazyme) on the QuantStudio Real-Time PCR Instrument (Applied biosystems, Foster City, CA, USA). The sequences of primers for qPCR were as follows: 5'-ATTTATCTCTTCCCCCAAAAG-3' (sense) and 5'-GCGGAGTGTGGACACATCT-3' (antisense) for SREBF2-AS1, 5'-CACCTCACTGCCCCATCTT-3' (sense) and 5'-TGAACGCCTTTTCTTGCTAA-3' (antisense) for SREBF2-AS1 (another primer pair), 5'-GCTGTAGTGAGATCCTGGT-3' (sense) and 5'-GAAACTTTGGGCAGCGACT-3' (antisense) for mutated SREBF2-AS1, 5'-GGCAAATCAAAAGAACAAGC-3' (sense) and 5'-AGAGTCAATGGAGTAGGGAGA-3' (antisense) for SREBF2, 5'-ATTTCTCCTATACTGTGGG-3' (sense) and 5'-ACTCTGGTTTGGGTTGTC-3' (antisense) for STARD4, 5'-GTCGGAGTCAACGGATTTG-3' (sense) and 5'-TGGGTGGAATCATATTGGAA-3' (antisense) for GAPDH. GAPDH served as the internal control. Relative expression was analyzed using the 2^−ΔΔCt^ method.

### Vectors construction, siRNAs and transfection

SREBF2-AS1 full-length sequences were PCR-amplified with the PrimeSTAR Max DNA Polymerase (cat. no. R045Q, Takara, Shiga, Japan) and the primers 5'-TAAACTTAAGCTTGGTACCACAAAAGCAAAATGGGAAAATGG-3' (sense) and 5'-TTTAAACGGGCCCTCTAGAGAGACAGAGTCTTGCTCTGTC-3' (antisense). The PCR products were subcloned into the *Kpn* I and *Xba* I sites of pcDNA3.1^(+)^ vector (cat. no. V79020, Invitrogen) with the NovoRec plus One step PCR Cloning Kit (cat. no. NR005, Novoprotein, Shanghai, China) to construct SREBF2-AS1 expression vector. m^6^A-modification sites (160, 2568 and 2607) mutated SREBF2-AS1 expression vector was constructed using the Fast Mutagenesis System (cat. no. FM111, TransGen, Beijing, China) with the primers 5'-TGTAGTGAGATCCTGGTCATGAAAGCAT-3' (sense) and 5'-ACCAGGATCTCACTACAGCCTTGCAG-3' (antisense) for the mutation of 160 site, 5'-CTAAAATTATGAGAGATCTGCAGCTGTT-3' (sense) and 5'-ATCTCTCATAATTTTAGGTTTCATCTTAAATG-3' (antisense) for the mutation of 2568 site, 5'-GAAATGATGACATGGTCAAAGGCCTC-3' (sense) and 5'-ACCATGTCATCATTTCCCCTTTGGC-3' (antisense) for the mutation of 2607 site.

Two pairs of cDNA oligonucleotides targeting SREBF2-AS1 were synthesized and subcloned into the shRNA lentivirus expressing vector LV-3 (cat. no. C06003, GenePharma, Shanghai, China), which was further used to generate shRNA lentivirus targeting SREBF2-AS1. Scrambled non-targeting shRNA lentivirus were used as negative control (NC). The sequences of shRNA oligonucleotides were as follows: 5'-GATCCGGATGTAGCCATCATACATGCTTCAAGAGAGCATGTATGATGGCTACATCCTTTTTTG-3' (sense) and 5'-AATTCAAAAAAGGATGTAGCCATCATACATGCTCTCTTGAAGCATGTATGATGGCTACATCCG-3' (antisense) for shRNA-SREBF2-AS1-1, 5'-GATCCGCAGCTCAGATTTGCATAGTGTTCAAGAGACACTATGCAAATCTGAGCTGCTTTTTTG-3' (sense) and 5'-AATTCAAAAAAGCAGCTCAGATTTGCATAGTGTCTCTTGAACACTATGCAAATCTGAGCTGCG-3' (anti-sense) for shRNA-SREBF2-AS1-2, 5'-GATCCGTTCTCCGAACGTGTCACGTTTCAAGAGAACGTGACACGTTCGGAGAACTTTTTTG-3' (sense) and 5'-AATTCAAAAAAGTTCTCCGAACGTGTCACGTTCTCTTGAAACGTGACACGTTCGGAGAACG-3' (antisense) for shRNA-NC.

METTL3 and METTL14 expression vectors were purchased from GenePharma. ON-TARGETplus Human METTL3 siRNA SMART Pool (cat. no. L-005170-02-0010), ON-TARGETplus Human METTL14 siRNA SMART Pool (cat. no. L-014169-02-0010), ON-TARGETplus Human FXR1 siRNA SMART Pool (cat. no. L-012011-00-0010), ON-TARGETplus Human TET1 siRNA SMART Pool (cat. no. L-014635-03-0010), and ON-TARGETplus Human SREBF2 siRNA SMART Pool (cat. no. L-009549-00-0010) were purchased from Horizon Discovery (Cambridge, England). Vectors and siRNAs transfection was conducted with the GP-transfect-Mate (GenePharma).

### Stable cell line construction

To construct HCC cells with stable overexpression of wild-type or m^6^A modification sites mutated SREBF2-AS1, SREBF2-AS1 expression vector or m^6^A-modification sites mutated SREBF2-AS1 expression vector was transfected into HuH-7 and HepG2 cells. Forty-eight hours later, the transfected cells were treated with 800 µg/ml G418 (cat. no. ant-gn-1, InvivoGen, San Diego, CA, USA) for four weeks to select wild-type or m^6^A modification sites mutated SREBF2-AS1 overexpressed HCC cells. To construct HCC cells with stable depletion of SREBF2-AS1, HuH-7 cells were infected with shRNA lentivirus targeting SREBF2-AS1. Ninety-six hours later, the infected cells were treated with 2 µg/ml puromycin (cat. no. ant-pr-1, InvivoGen) for four weeks to select SREBF2-AS1 stably depleted HuH-7 cells.

### Western blot

Total proteins were collected from indicated cells using RIPA buffer (Beyotime, Shanghai, China). The proteins were separated by sodium dodecyl sulfate polyacrylamide gel electrophoresis. The gels were cut according to the molecular weight of target proteins, followed by being transferred to polyvinylidene fluoride membranes (Millipore, Billerica, MA, USA). After blocking, the membranes were incubated with primary antibodies against METTL3 (cat. no. 86132, 1:1000, Cell Signaling Technology, Danvers, MA, USA), METTL14 (cat. no. 48699, 1:1000, Cell Signaling Technology), SREBF2 (cat. no. ab30682, 1:500, Abcam, Cambridge, MA, USA) or GAPDH (cat. no. ab8245, 1:10000, Abcam). After three washes, the membranes were incubated with second antibody and scanned on an Odyssey infrared scanner (Li-Cor, Lincoln, NE, USA).

### Cell proliferation, apoptosis, migration, and viability assays

Cell proliferation was evaluated by the Cell Counting Kit-8 (CCK-8) and 5-ethynyl-2'-deoxyuridine (EdU) incorporation assays as we previously described^23^. For CCK-8 assay, 2000 indicated HCC cells per well were plated into 96-well plate. After culture for indicated time, CCK-8 reagent (cat. no. CK04, Dojindo, Shanghai, China) was added to each well to detect cell proliferation strictly following the provided protocol. EdU incorporation assay was performed using the Cell-Light EdU Apollo567 In Vitro Kit (cat. no. C10310-1, RiboBio, Guangzhou, China). Cell apoptosis was evaluated by the terminal deoxynucleotidyl transferase (TdT)-mediated dUTP nick end labeling (TUNEL) assay using the One Step TUNEL Apoptosis Assay Kit (cat. no. C1090, Beyotime) following the provided protocol. Cell migration was evaluated by transwell migration assay as previously described^43–46^. Cell viability was evaluated by the Glo cell viability assay using the CellTiter-Glo Luminescent Cell Viability Assay (cat. no. G7570, Promega, Madison, WI, USA) as previously described^47^.

### Chromatin isolation by RNA purification (ChIRP) assay

ChIRP assay was performed in HuH-7 cells using the EZ-Magna ChIRP RNA Interactome Kit (cat. no. 17-10495, Millipore). The sequences of SREBF2-AS1 antisense probes were as follows: 1, 5'-ctcttttgggggaagagata-3'; 2, 5'-gtcacgcttagagagcttag-3'; 3, 5'-gaaactttgggcagcgactg-3'; 4, 5'-tggtctggagaccatggaga-3'; 5, 5'-cgggcgcaacgcaaacatgg-3'; 6, 5'-aatctgagctgctgatcgat-3'; 7, 5'-cagcggctcctttaaacaag-3'; 8, 5'-taggcagctgggaagatgac-3'; 9, 5'-aatctgcaaccttgtcaagc-3'; 10, 5'-cagtgaggtgcttgaaggag-3'; 11, 5'-gatcactaagcaacagctgc-3'. The enrichment of DNA was detected by qPCR with the primers: 5'-GGGGGAGGGACCTCACTAT-3' (sense) and 5'-AATGGGACCAGGCTCATCTC-3' (antisense) for *SREBF2* promoter; 5'-ACGCACGCAGTACAATCT-3' (sense) and 5'-CACTCATAAAAACGAGGGA-3' (antisense) for *SREBF2* gene body; 5'-TACCCCATCTCCTACCTC-3' (sense) and 5'-TAATACCCCAACAGACCAA-3' (antisense) for *SREBF2* 3’UTR region; 5'-GGCTACTAGCGGTTTTACGG-3' (sense) and 5'-CGAACAGGAGGAGCAGAGA-3' (antisense) for *GAPDH* promoter.

### RNA immunoprecipitation (RIP) and methylated RNA immunoprecipitation (MeRIP) assays

RIP assay was performed in indicated cells using the EZ-Magna RIP Kit (cat. no. 17-701, Millipore) and the primary antibodies against FXR1 (cat. no. 03-176, Millipore). The enrichment of SREBF2-AS1 was detected using qPCR as above described. m^6^A modification level of SREBF2-AS1 in indicated tissues and cells were detected by the MeRIP assay using the Magna MeRIP m^6^A Kit (cat. no. 17-10499, Millipore). Enriched m^6^A-modified SREBF2-AS1 was quantified by RT-qPCR as above described and normalized to total RNA.

### CUT&RUN assay

CUT&RUN assay was performed in indicated cells using the CUT&RUN Assay Kit (cat. no. #86652, Cell Signaling Technology) and the primary antibodies against FXR1 (cat. no. 03-176, Millipore) and TET1 (cat. no. #71128, Cell Signaling Technology). The enrichment of *SREBF2* promoter was detected using qPCR with the primers: 5'-GGGGGAGGGACCTCACTAT-3' (sense) and 5'-AATGGGACCAGGCTCATCTC-3' (antisense).

### Bisulfite DNA sequencing

DNA was extracted from indicated cells with the TIANamp Genomic DNA Kit (cat. no. DP304, TIANGEN, Beijing, China) and bisulfite-treated with the EZ DNA Methylation-Gold Kit (cat. no. D5005, Zymo Research, Irvine, CA, USA). Modified genomic DNA was PCR-amplified with the primers 5’-GTTAATTTTTTATTTTTAGGTTAGTGGA-3’ (sense) and 5’-TAAAACCAAACTCATCTCAACCAA-3’ (antisense). The PCR products were subcloned into the T-Vector pMD™19 (cat. no. 3271, Takara, Dalian, China) and transformed into Escherichia coli. Candidate clones were sequenced by Sangon Biotech (Shanghai, China).

### Statistical analysis

All statistical analyses were conducted using the GraphPad Prism 6.0 Software. Student’s *t*-test, one-way ANOVA followed by Dunnett's multiple comparisons test, Mann–Whitney test, Wilcoxon matched-pairs signed rank test, log-rank test, Pearson chi-square test, and Spearman correlation analysis were performed as indicated in the figure and table legends. *p* < 0.05 was considered as statistically significant.

### Ethical approval

Human specimens were collected from Affiliated Hospital of Youjiang Medical University for Nationalities. Written informed consents were signed by all participants. This research was conducted following the Declaration of Helsinki and reviewed and approved by Affiliated Hospital of Youjiang Medical University for Nationalities Institutional Review Board.

## Results

### SREBF2-AS1 is highly expressed in HCC and correlated with poor prognosis of HCC patients

**Figure 1:**
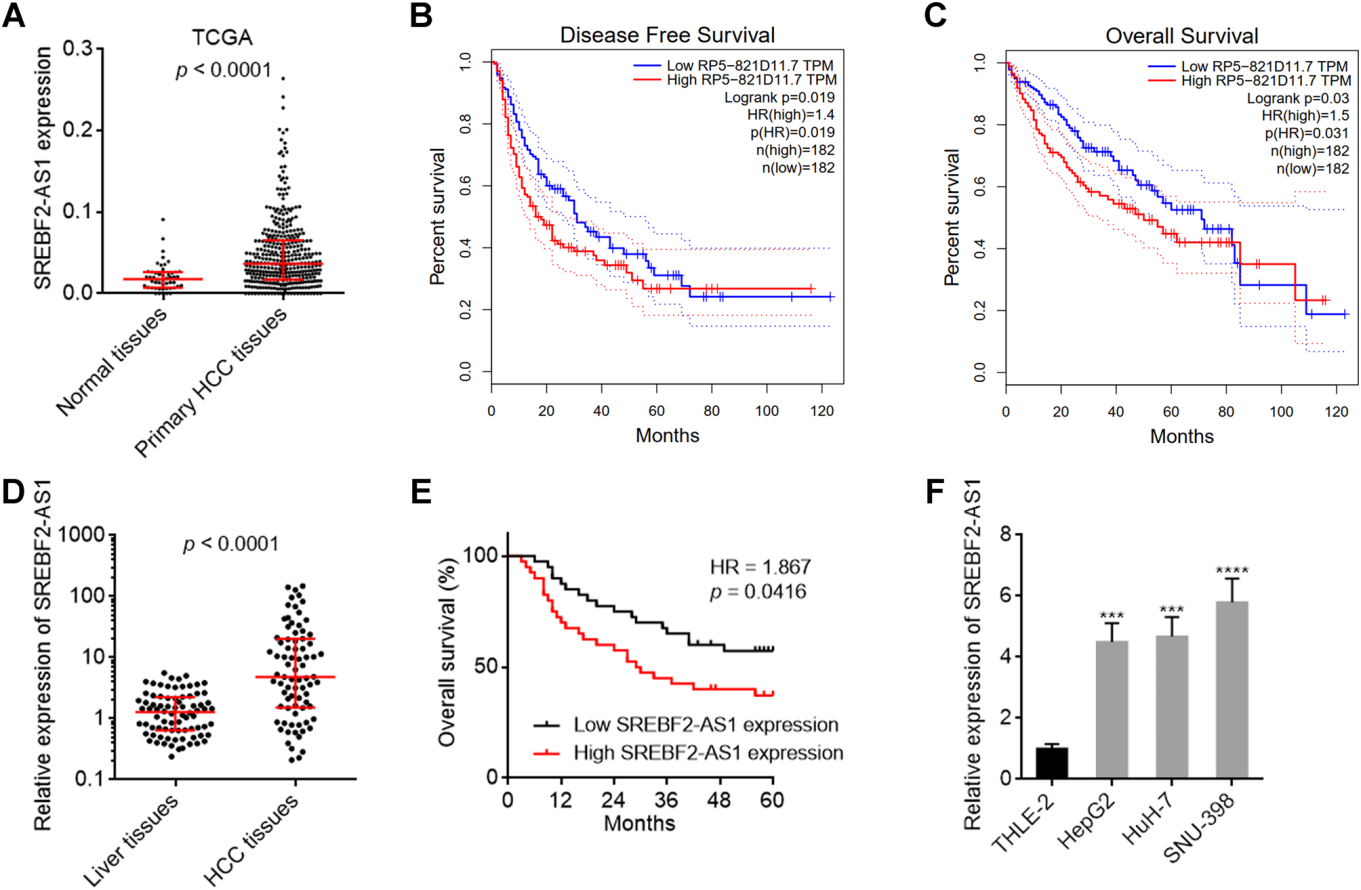
Expression pattern and prognostic correlation of SREBF2-AS1 in HCC. (**A**) SREBF2-AS1 expression in 50 normal liver tissues and 371 HCC tissues, derived from TCGA LIHC dataset. *p* < 0.0001 by Mann–Whitney test. (**B**) The correlation between SREBF2-AS1 (RP5-821D11.7) expression and disease-free survival of HCC patients, derived from TCGA LIHC dataset, analysed by the online tool GEPIA. **(C**) The correlation between SREBF2-AS1 (RP5-821D11.7) expression and overall survival of HCC patients, derived from TCGA LIHC dataset, analysed by the online tool GEPIA. (**D**) SREBF2-AS1 expression in 80 pairs of HCC tissues and matched noncancerous liver tissues was measured by RT-qPCR. *p* < 0.0001 by Wilcoxon matched-pairs signed rank test. (**E**) Kaplan–Meier survival analysis of the correlation between SREBF2-AS1 expression and overall survival of HCC patients. Median SREBF2-AS1 expression level was used as cut-off. n = 80, HR = 1.867, *p* = 0.0416 by log-rank test. (**F**) SREBF2-AS1 expression in immortalized liver cell line THLE-2 and HCC cell lines HepG2, HuH-7, and SNU-398 was measured by RT-qPCR. Results are shown as mean ± standard deviation (SD) of 3 independent experiments. ***p < 0.001, ****p < 0.0001 by one-way ANOVA followed by Dunnett's multiple comparisons test.

The expression of SREBF2-AS1 in primary HCC and normal liver tissues was analyzed using The Cancer Genome Atlas (TCGA) Liver Hepatocellular Carcinoma (LIHC) data. The results showed that SREBF2-AS1 was significantly highly expressed in HCC tissues, compared with normal liver tissues (Fig. 1A). Analyses of the correlation between SREBF2-AS1 expression level and clinicopathological characteristics of HCC patients based on TCGA LIHC dataset showed that high expression of SREBF2-AS1 was positively correlated with high alpha-fetoprotein (AFP) concentration and advanced stage (Table 1). Furthermore, analyses of the correlation between SREBF2-AS1 expression and clinical prognoses of HCC patients based on TCGA LIHC dataset by the online tool GEPIA (Gene Expression Profiling Interactive Analysis, http://gepia.cancer-pku.cn/) showed that high expression of SREBF2-AS1 was positively associated with poor disease-free survival and overall survival (Fig. 1B,C). To further confirm the clinical significances of SREBF2-AS1 in HCC, we measured SREBF2-AS1 expression in 80 pairs of HCC tissues and matched noncancerous liver tissues. The results showed that SREBF2-AS1 was highly expressed in HCC tissues compared with noncancerous liver tissues (Fig. 1D). Kaplan–Meier survival analysis also showed that high expression of SREBF2-AS1 was associated with poor overall survival (Fig. 1E). Moreover, SREBF2-AS1 was highly expressed in HCC cells HepG2, HuH-7, and SNU-398 compared with immortalized noncancerous liver cells THLE-2 (Fig. 1F).

**Table 1:** Correlation between SREBF2-AS1 expression levels and clinicopathological characteristics of HCC patients according to TCGA dataset.

Feature	SREBF2-AS1 expression		χ^2^	*p* value*
	Low	High^a^		
Age			1.040	0.308
> 50	142	150		
≤ 50	43	35		
Gender			1.397	0.237
Male	130	120		
Female	55	66		
Child–Pugh			1.048	0.592
A	109	108		
B	10	11		
C	1	0		
AFP (ng/ml)			5.742	**0.017**
> 20	56	75		
≤ 20	84	63		
Grade			3.814	0.051
G1–G2	125	107		
G3–G4	58	76		
Tumor pathologic pt			7.281	**0.007**
T1–T2	148	127		
T3–T4	35	58		
Vascular invasion			0.576	0.750
None	109	97		
Micro	47	46		
Macro	7	9		
TNM staging			7.648	**0.006**
I–II	139	118		
III–IV	33	56		

^a^The medium expression level was used as the cutoff.

*p value was acquired by Pearson chi-square tests.

### m^6^A modification level of SREBF2-AS1 is increased in HCC and correlated with poor prognosis of HCC patients

**Figure 2:**
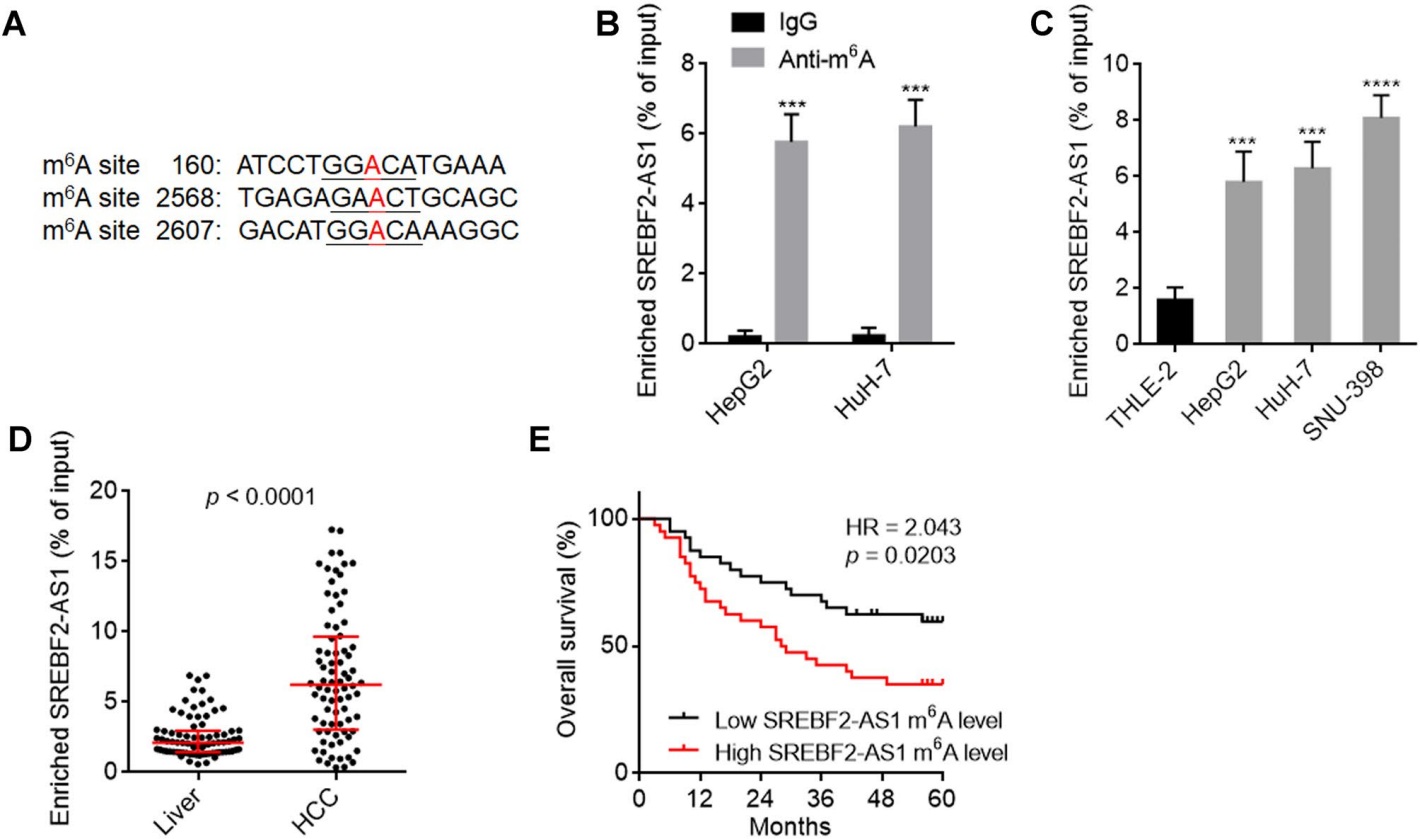
m^6^A modification level of SREBF2-AS1 in HCC and its correlation with prognosis of HCC patients. (**A**) Predicted three m^6^A modification sites on SREBF2-AS1. (**B**) m^6^A-modified SREBF2-AS1 was detected in HepG2 and HuH-7 cells using MeRIP assays. (**C**) m^6^A modification level of SREBF2-AS1 in immortalized liver cell line THLE-2 and HCC cell lines HepG2, HuH-7, and SNU-398 was measured by MeRIP assays. (**D**) m^6^A modification level of SREBF2-AS1 in 80 pairs of HCC tissues and matched noncancerous liver tissues was measured by MeRIP assays. *p* < 0.0001 by Wilcoxon matched-pairs signed rank test. (**E**) Kaplan–Meier survival analysis of the correlation between SREBF2-AS1 m^6^A modification level and overall survival of HCC patients. Median SREBF2-AS1 m^6^A modification level was used as cut-off. n = 80, HR = 2.043, *p* = 0.0203 by log-rank test. For (**B**) and (**C**), results are shown as mean ± SD of 3 independent experiments. ***p < 0.001, ****p < 0.0001 by Student’s *t* test (**B**) or one-way ANOVA followed by Dunnett's multiple comparisons test (**C**).

The online tool SRAMP (sequence-based RNA adenosine methylation site predictor, http://www.cuilab.cn/sramp) predicted three m^6^A modification sites on SREBF2-AS1 (Fig. 2A). MeRIP assays confirmed the existence of m^6^A-modified SREBF2-AS1 in HepG2 and HuH-7 cells (Fig. 2B and Supplementary Fig. S1A). MeRIP assays further showed that the m^6^A modification level of SREBF2-AS1 was elevated in HCC cells HepG2, HuH-7, and SNU-398 compared with immortalized liver cells THLE-2 (Fig. 2C and Supplementary Fig. S1B). In our HCC cohort containing 80 pairs of HCC tissues and matched noncancerous liver tissues, MeRIP assays were undertaken to measure m^6^A modification level of SREBF2-AS1, and the results showed that the m^6^A modification level of SREBF2-AS1 was increased in HCC tissues compared with noncancerous liver tissues (Fig. 2D). Similar with SREBF2-AS1 expression level, high m^6^A modification level of SREBF2-AS1 was also associated with poor overall survival of HCC patients (Fig. 2E). These data suggested that m^6^A modification level of transcript may be prognostic biomarker for HCC.

### METTL3 and METTL14-mediated m^6^A modification upregulates SREBF2-AS1 expression through increasing SREBF2-AS1 transcript stability

**Figure 3:**
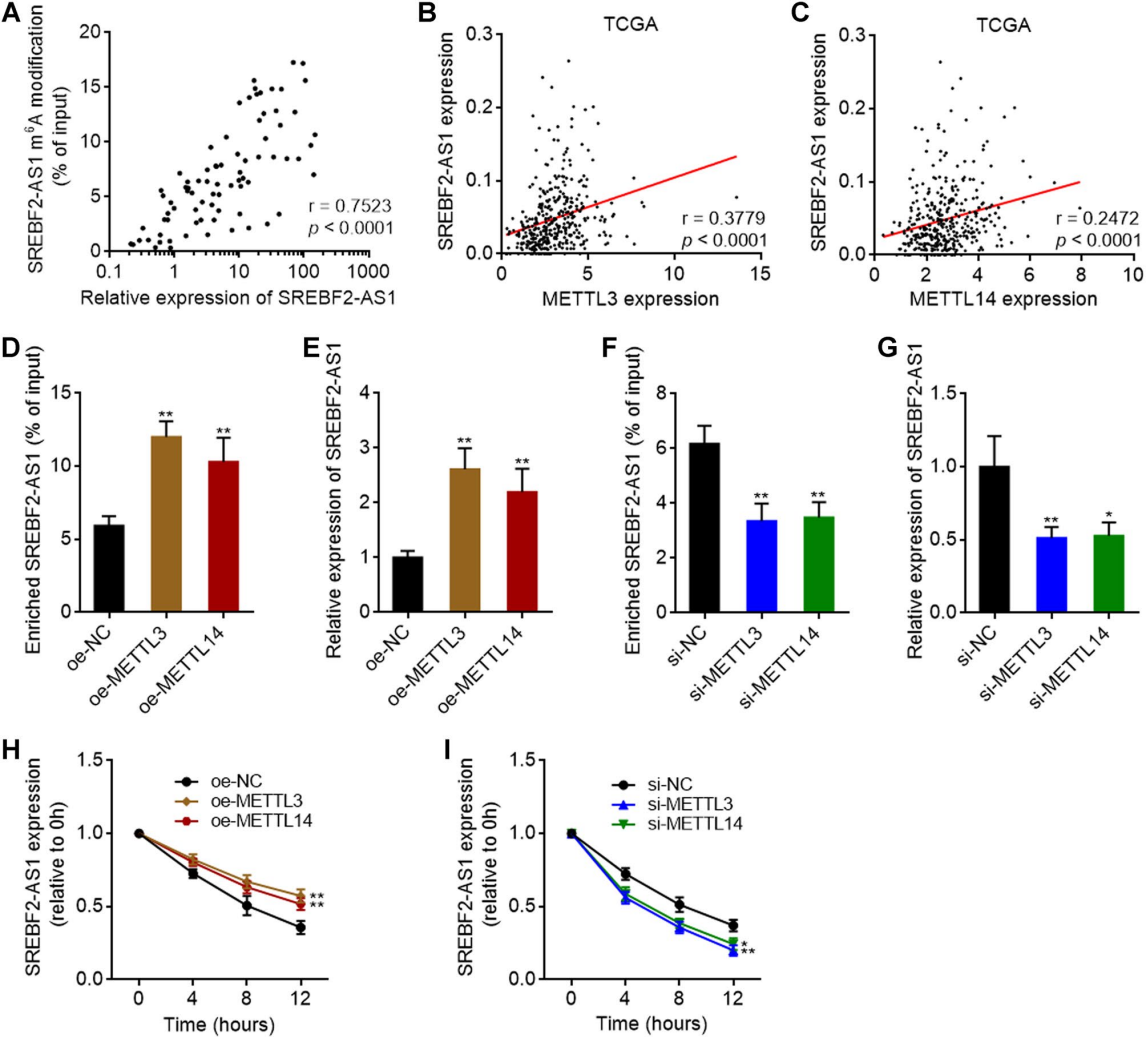
METTL3 and METTL14-mediated m^6^A modification upregulated SREBF2-AS1 expression via increasing SREBF2-AS1 transcript stability. (**A**) The correlation between expression level and m^6^A modification level of SREBF2-AS1 in 80 HCC tissues. r = 0.7523, *p* < 0.0001 by Spearman correlation analysis. (**B**) The correlation between SREBF2-AS1 and METTL3 expression level in 371 HCC tissues, derived from TCGA LIHC dataset. r = 0.3779, *p* < 0.0001 by Spearman correlation analysis. (**C**) The correlation between SREBF2-AS1 and METTL14 expression level in 371 HCC tissues, derived from TCGA LIHC dataset. r = 0.2472, *p* < 0.0001 by Spearman correlation analysis. (**D**) m^6^A modification level of SREBF2-AS1 in HuH-7 cells with METTL3 or METTL14 overexpression was measured by MeRIP assays. (**E**) SREBF2-AS1 expression in HuH-7 cells with METTL3 or METTL14 overexpression was measured by RT-qPCR. **(F**) m^6^A modification level of SREBF2-AS1 in HuH-7 cells with METTL3 or METTL14 depletion was measured by MeRIP assays. (**G**) SREBF2-AS1 expression in HuH-7 cells with METTL3 or METTL14 depletion was measured by RT-qPCR. (**H**,**I**) SREBF2-AS1 transcript stability in HuH-7 cells with METTL3 or METTL14 overexpression (**H**) or depletion (**I**) over time was measured after blocking new RNA synthesis with α‑amanitin (50 µM). For (**D**–**I**), results are shown as mean ± SD of 3 independent experiments. *p < 0.05, **p < 0.01 by one-way ANOVA followed by Dunnett's multiple comparisons test.

Next, we investigated whether m^6^A modification regulates SREBF2-AS1 expression. The correlation between SREBF2-AS1 expression level and m^6^A modification level was analyzed in HCC tissues, and the results showed that m^6^A modification level of SREBF2-AS1 was positively correlated with expression level of SREBF2-AS1 in HCC tissues (Fig. 3A). To further identify the writers responsible for the installation of m^6^A on SREBF2-AS1, we analyzed the expression correlation between SREBF2-AS1 and writers in HCC tissues based on TCGA LIHC dataset. The results showed that the expression of METTL3 and METTL14, but not METTL16, was positively correlated with the expression of SREBF2-AS1 in HCC tissues (Fig. 3B,C and Supplementary Fig. S2A). MeRIP assays showed that ectopic expression of METTL3 or METTL14 both increased m^6^A modification level and expression level of SREBF2-AS1 (Fig. 3D,E and Supplementary Fig. S2B–E). Conversely, depletion of METTL3 or METTL14 both reduced m^6^A modification level and expression level of SREBF2-AS1 (Fig. 3F,G and Supplementary Fig. S2F–I). To investigate whether m^6^A-induced transcript stability is responsible for the upregulation of SREBF2-AS1 expression, METTL3 or METTL14 overexpressed or silenced HuH-7 cells were treated with α‑amanitin to block new RNA generation. Then, the degradation of SREBF2-AS1 transcript was measured. The results showed that ectopic expression of METTL3 or METTL14 elongated the half-life of SREBF2-AS1 transcript (Fig. 3H), and while depletion of METTL3 or METTL14 shortened the half-life of SREBF2-AS1 transcript (Fig. 3I).

### SREBF2-AS1 promotes HCC progression and sorafenib resistance in an m^6^A-dependent manner

**Figure 4:**
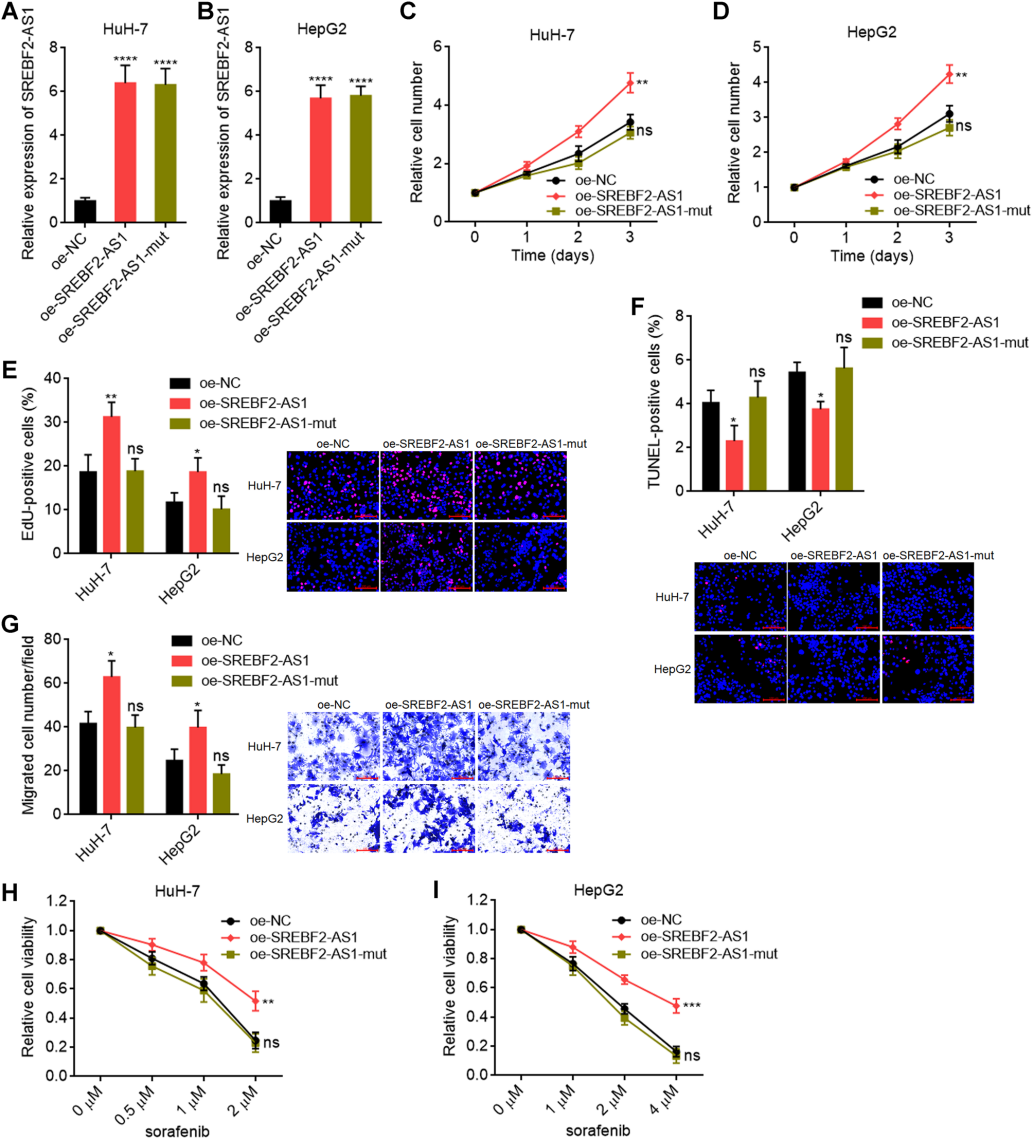
SREBF2-AS1 enhanced oncogenic properties and sorafenib resistance of HCC cells in an m^6^A-dependent manner. (**A**,**B**) SREBF2-AS1 expression was measured by RT-qPCR in HuH-7 (**A**) or HepG2 (**B**) cells with stable overexpression of wild-type or m^6^A modification sites mutated SREBF2-AS1. (**C**,**D**) Cellular proliferation of HuH-7 (**C**) or HepG2 (**D**) cells with overexpression of wild-type or m^6^A modification sites mutated SREBF2-AS1 was measured by CCK-8 assays. (**E**) Cellular proliferation of HuH-7 and HepG2 cells with overexpression of wild-type or m^6^A modification sites mutated SREBF2-AS1 was measured by EdU incorporation assays. Scale bars, 100 µm. (**F**) Cellular apoptosis of HuH-7 and HepG2 cells with overexpression of wild-type or m^6^A modification sites mutated SREBF2-AS1 was measured by TUNEL assays. Scale bars, 100 µm. (**G**) Cellular migration of HuH-7 and HepG2 cells with overexpression of wild-type or m^6^A modification sites mutated SREBF2-AS1 was measured by transwell migration assays. Scale bars, 100 µm. (**H**,**I**) Cell viability was measured by Glo cell viability assays in HuH-7 (**H**) or HepG2 (**I**) cells with overexpression of wild-type or m^6^A modification sites mutated SREBF2-AS1 after sorafenib treatment, normalized to no sorafenib treatment. Results are shown as mean ± SD of 3 independent experiments. *p < 0.05, **p < 0.01, ***p < 0.001, ****p < 0.0001, ns, not significant, by one-way ANOVA followed by Dunnett's multiple comparisons test.

Next, we investigated the potential roles of SREBF2-AS1 in HCC. We constructed HuH-7 and HepG2 cells with stable overexpression of wild-type or three predicted m^6^A modification sites-mutated SREBF2-AS1 (Fig. 4A, B and Supplementary Fig. S3A, B). The mutation of these three m^6^A modification sites almost completely abolished m^6^A modification on SREBF2-AS1 (Supplementary Fig. S3C, D). CCK-8 assays showed that ectopic expression of SREBF2-AS1 promoted cell proliferation of both HuH-7 and HepG2 cells, which was abolished by the mutation of m^6^A modification sites on SREBF2-AS1 (Fig. 4C,D). EdU incorporation assays further confirmed the pro-proliferative roles of wild-type SREBF2-AS1, but not m^6^A modification sites-mutated SREBF2-AS1 (Fig. 4E). TUNEL assays showed that ectopic expression of SREBF2-AS1 repressed cell apoptosis of both HuH-7 and HepG2 cells, which was also abolished by the mutation of m^6^A modification sites on SREBF2-AS1 (Fig. 4F). Transwell assays showed that ectopic expression of SREBF2-AS1 promoted cell migration of both HuH-7 and HepG2 cells, which was abolished by the mutation of m^6^A modification sites on SREBF2-AS1 (Fig. 4G). Cell viabilities were measured in HuH-7 and HepG2 cells with stable overexpression of wild-type or m^6^A modification sites-mutated SREBF2-AS1 after sorafenib treatment. The results showed that ectopic expression of wild-type SREBF2-AS1, but not m^6^A modification sites-mutated SREBF2-AS1 promoted sorafenib resistance of both HuH-7 and HepG2 cells (Fig. 4H,I). These data suggested that SREBF2-AS1 promoted HCC progression and sorafenib resistance in an m^6^A-dependent manner.

To further confirm the functions of SREBF2-AS1 in HCC, we constructed HuH-7 cells with stable depletion of SREBF2-AS1 (Supplementary Fig. S4A, B). CCK-8 and EdU incorporation assays showed that depletion of SREBF2-AS1 inhibited cell proliferation of HuH-7 cells (Supplementary Fig. S4C, D). TUNEL assays showed that depletion of SREBF2-AS1 promoted cell apoptosis of HuH-7 cells (Supplementary Fig. S4E). Transwell migration assays showed that depletion of SREBF2-AS1 repressed cell migration of HuH-7 cells (Supplementary Fig. S4F). Cell viabilities measurement further showed that depletion of SREBF2-AS1 enhanced sorafenib sensitivity of HuH-7 cells (Supplementary Fig. S4G).

### SREBF2-AS1 upregulates *SREBF2* transcription in an m^6^A-depedent manner

**Figure 5:**
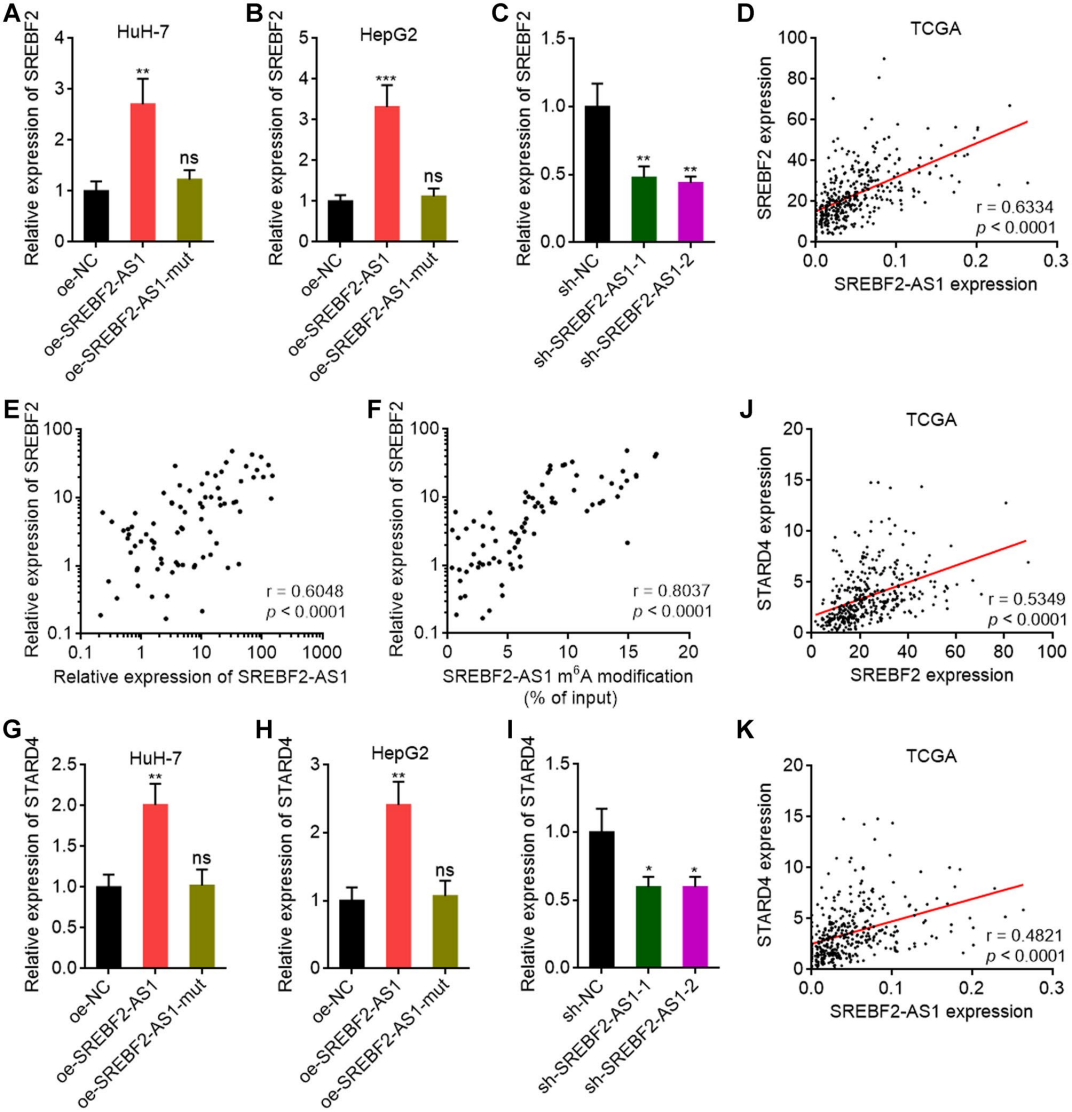
SREBF2-AS1 upregulated *SREBF2* transcription in an m^6^A-dependent manner. (**A**,**B**) SREBF2 expression was measured by RT-qPCR in HuH-7 (**A**) or HepG2 (**B**) cells with stable overexpression of wild-type or m^6^A modification sites mutated SREBF2-AS1. (**C**) SREBF2 expression was measured by RT-qPCR in HuH-7 cells with stable depletion of SREBF2-AS1. (**D**) The correlation between SREBF2 and SREBF2-AS1 expression level in 371 HCC tissues, derived from TCGA LIHC dataset. r = 0.6334, *p* < 0.0001 by Spearman correlation analysis. (**E**) The correlation between SREBF2 and SREBF2-AS1 expression level in 80 HCC tissues. r = 0.6048, *p* < 0.0001 by Spearman correlation analysis. (**F**) The correlation between SREBF2 expression level and m^6^A modification level of SREBF2-AS1 in 80 HCC tissues. r = 0.8037, *p* < 0.0001 by Spearman correlation analysis. (**G**,**H**) STARD4 expression was measured by RT-qPCR in HuH-7 (**G**) or HepG2 (**H**) cells with stable overexpression of wild-type or m^6^A modification sites mutated SREBF2-AS1. (**I**) STARD4 expression was measured by RT-qPCR in HuH-7 cells with stable depletion of SREBF2-AS1. (**J**) The correlation between STARD4 and SREBF2 expression level in 371 HCC tissues, derived from TCGA LIHC dataset. r = 0.5349, *p* < 0.0001 by Spearman correlation analysis. (**K**) The correlation between STARD4 and SREBF2-AS1 expression level in 371 HCC tissues, derived from TCGA LIHC dataset. r = 0.4821, *p* < 0.0001 by Spearman correlation analysis. For (**A**–**C**) and (**G**–**I**), results are shown as mean ± SD of 3 independent experiments. *p < 0.05, **p < 0.01, ***p < 0.001, ns, not significant, by one-way ANOVA followed by Dunnett's multiple comparisons test.

SREBF2-AS1 is the antisense strand RNA of *SREBF2*. Several antisense strand RNAs have been reported to regulate their sense strand genes expression^48,49^. Furthermore, SREBF2 has been frequently reported to modulate cell proliferation, migration, and drug resistance of HCC cells^50–54^. Thus, we further investigated whether SREBF2 is a downstream target of SREBF2-AS1 and the functional mediator of SREBF2-AS1 in HCC. Intriguingly, we found that SREBF2 was upregulated in HuH-7 and HepG2 cells with stable overexpression of wild-type SREBF2-AS1, but not m^6^A modification sites-mutated SREBF2-AS1 (Fig. 5A,B and Supplementary Fig. S5A). Conversely, depletion of SREBF2-AS1 decreased SREBF2 expression, which was rescued by ectopic expression of SREBF2-AS1 (Fig. 5C and Supplementary Fig. S5B, C). The expression of SREBF2 was positively correlated with SREBF2-AS1 in HCC tissues, based on TCGA LIHC dataset (Fig. 5D). The positive correlation between SREBF2-AS1 and SREBF2 expression in HCC tissues was further verified in our HCC cohort (Fig. 5E). Moreover, the expression level of SREBF2 was also positively correlated with m^6^A modification level of SREBF2-AS1 in HCC tissues (Fig. 5F). SREBF2 has been reported to promote the transcription of *STARD4*, which is a critical functional mediator of SREBF2 in HCC^50^. Here, we further found that consistent with SREBF2, STARD4 was upregulated in HuH-7 and HepG2 cells with overexpression of wild-type SREBF2-AS1, but not m^6^A modification sites-mutated SREBF2-AS1 (Fig. 5G,H). Depletion of SREBF2-AS1 also decreased STARD4 expression, which was rescued by ectopic expression of SREBF2-AS1 (Fig. 5I and Supplementay Fig. S5D). The expression of STARD4 was positively correlated with SREBF2 and SREBF2-AS1 in HCC tissues, based on TCGA LIHC dataset (Fig. 5J,K), further supporting the positive regulation axis of SREBF2-AS1/SREBF2/STARD4.

### m^6^A-modified SREBF2-AS1 induces DNA demethylation at *SREBF2* promoter through recruiting FXR1 and TET1

**Figure 6:**
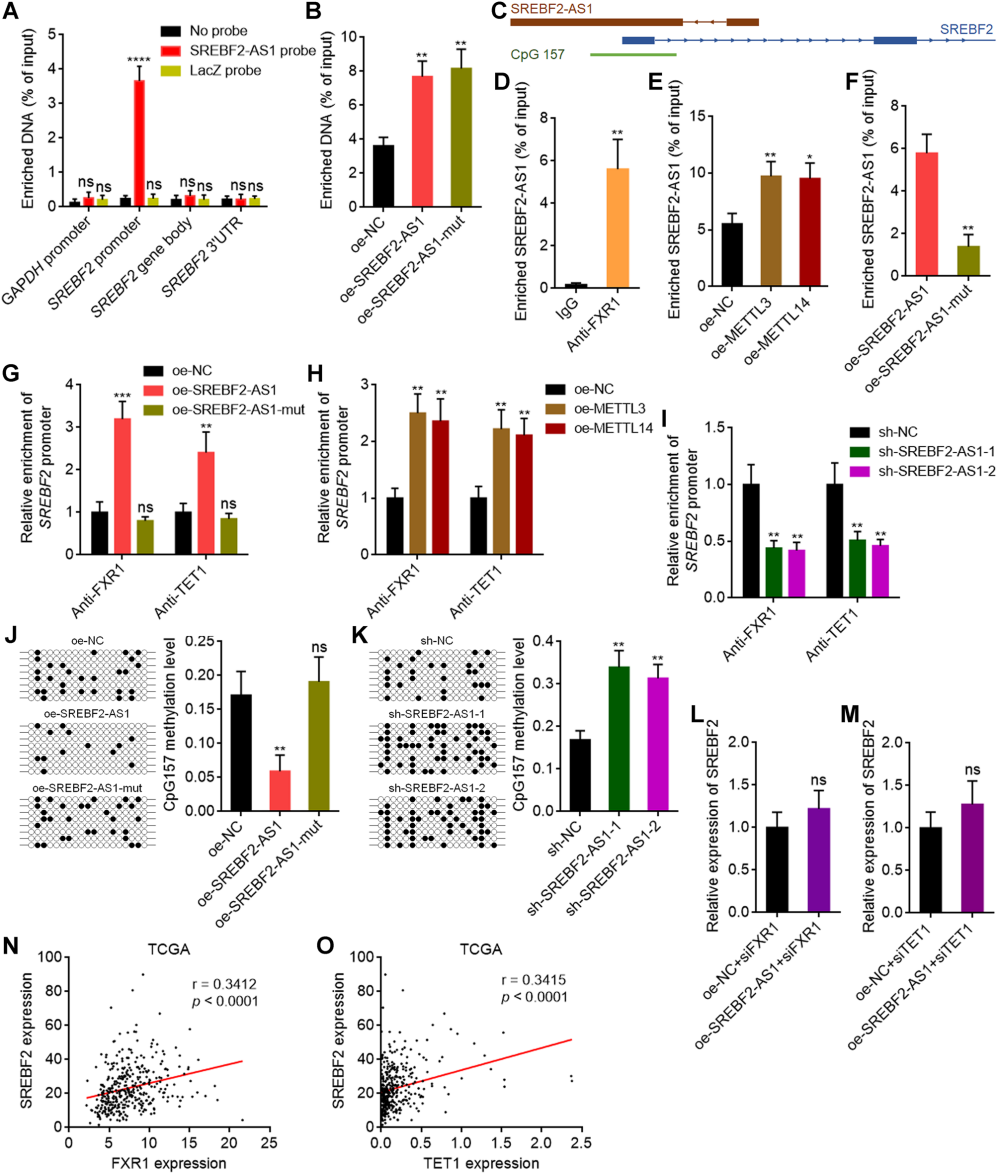
m^6^A-modified SREBF2-AS1 induced DNA demethylation at *SREBF2* promoter through recruiting FXR1 and TET1. (**A**) The binding of SREBF2-AS1 to *SREBF2* promoter, gene body, or 3’UTR in HuH-7 cells was measured using ChIRP assays with SREBF2-AS1 antisense probes or control probes. *GAPDH* promoter was employed as negative control. (**B**) ChIRP assays with SREBF2-AS1 antisense probes were performed in HuH-7 cells with overexpression of wild-type or m^6^A modification sites mutated SREBF2-AS1 to measure the binding of SREBF2-AS1 to *SREBF2* promoter. (**C**) Schematic structure of *SREBF2-AS1* and *SREBF2* genomic locus. (**D**) The binding of SREBF2-AS1 to FXR1 in HuH-7 cells was measured using RIP assays with FXR1 specific antibody. (**E**) RIP assays with FXR1 specific antibody were performed in HuH-7 cells with METTL3 or METTL14 overexpression to measure the binding of SREBF2-AS1 to FXR1. (**F**) RIP assays with FXR1 specific antibody were performed in HuH-7 cells with wild-type or m^6^A modification sites mutated SREBF2-AS1 overexpression to measure the binding of SREBF2-AS1 to FXR1. (**G**) CUT&RUN assays with FXR1 or TET1 specific antibodies were performed in HuH-7 cells with wild-type or m^6^A modification sites mutated SREBF2-AS1 overexpression to measure the binding of FXR1 and TET1 to *SREBF2* promoter. **(H**) CUT&RUN assays with FXR1 or TET1 specific antibodies were performed in HuH-7 cells with METTL3 or METTL14 overexpression to measure the binding of FXR1 and TET1 to *SREBF2* promoter. (**I**) CUT&RUN assays with FXR1 or TET1 specific antibodies were performed in HuH-7 cells with SREBF2-AS1 depletion to measure the binding of FXR1 and TET1 to *SREBF2* promoter. (**J**) Bisulfate DNA sequencing of CpG157 from HuH-7 cells with wild type or m^6^A modification sites mutated SREBF2-AS1 overexpression. (**K**) Bisulfate DNA sequencing of CpG157 from HuH-7 cells with SREBF2-AS1 depletion. (**L**) SREBF2 expression was measured by RT-qPCR in HuH-7 cells with SREBF2-AS1 overexpression and concurrent FXR1 depletion. (**M**) SREBF2 expression was measured by RT-qPCR in HuH-7 cells with SREBF2-AS1 overexpression and concurrent TET1 depletion. (**N**) The correlation between SREBF2 and FXR1 expression level in 371 HCC tissues, derived from TCGA LIHC dataset. r = 0.3412, *p* < 0.0001 by Spearman correlation analysis. **(O**) The correlation between SREBF2 and TET1 expression level in 371 HCC tissues, derived from TCGA LIHC dataset. r = 0.3415, *p* < 0.0001 by Spearman correlation analysis. For (**A**,**B**), and (**D**–**M**), results are shown as mean ± SD of 3 independent experiments. *p < 0.05, **p < 0.01, ***p < 0.001, ****p < 0.0001, ns, not significant, by one-way ANOVA followed by Dunnett's multiple comparisons test (**A**,**B**,**E**,**G**–**K**) or Student’s *t* test (**D**,**F**,**L**,**M**).

Given that SREBF2-AS1 is an antisense RNA of *SREBF2* and upregulates *SREBF2* transcription, we next investigated whether SREBF2-AS1 directly bound to *SREBF2* using ChIRP assays. The results revealed that *SREBF2* promoter region, but not *SREBF2* gene body or 3’UTR region, was specifically enriched in SREBF2-AS1 probe group (Fig. 6A), suggesting that SREBF2-AS1 directly bound to *SREBF2* promoter region. Ectopic expression of wild-type or m^6^A modification sites-mutated SREBF2-AS1 both lead to more enrichment of *SREBF2* promoter by SREBF2-AS1 probe (Fig. 6B), suggesting that not only wild-type, but also m^6^A modification sites-mutated SREBF2-AS1 both binds to *SREBF2* promoter. However, our above results have shown that only wild-type, but not m^6^A modification sites-mutated SREBF2-AS1 upregulated *SREBF2* transcription. Analyses of the genomic structure of *SREBF2*, we noted that *SREBF2* promoter region is located at a CpG island CpG157 (Fig. 6C). Recently, m^6^A-modified RNA was reported to induce DNA demethylation via recruiting m^6^A reader FXR1 and DNA 5-methylcytosine dioxygenase TET1^55^. Thus, we hypothesized that m^6^A-modified SREBF2-AS1 modulates *SREBF2* transcription in such a manner. RIP assays showed that SREBF2-AS1 bound to FXR1 (Fig. 6D). Overexpression of METTL3 or METTL14 promoted the interaction between SREBF2-AS1 and FXR1 (Fig. 6E). Mutation of m^6^A modification sites on SREBF2-AS1 significantly decreased the binding of SREBF2-AS1 to FXR1 (Fig. 6F). These data suggested that SREBF2-AS1 specifically binds to FXR1 in an m^6^A-depedent manner. CUT&RUN assays showed that overexpression of wild-type, but not m^6^A modification sites-mutated SREBF2-AS1 promoted the binding of FXR1 and TET1 to *SREBF2* promoter (Fig. 6G). Induction of m^6^A modification on SREBF2-AS1 by METLL3 or METTL14 overexpression also promoted the binding of FXR1 and TET1 to *SREBF2* promoter (Fig. 6H). Depletion of SREBF2-AS1 reduced the binding of FXR1 and TET1 to *SREBF2* promoter (Fig. 6I). These data suggested that SREBF2-AS1 binds and recruits FXR1 and TET1 to *SREBF2* promoter in an m^6^A-depedent manner. Bisulfate sequencing showed that ectopic expression of wild-type, but not m^6^A modification sites-mutated SREBF2-AS1 induced DNA demethylation of CpG157 (Fig. 6J). Conversely, depletion of SREBF2-AS1 upregulated CpG157 DNA methylation level (Fig. 6K). Depletion of FXR1 or TET1 both abolished the upregulation of *SREBF2* transcription caused by SREBF2-AS1 overexpression (Fig. 6L, M), suggesting that FXR1 and TET1 were critical mediators of the effects of SREBF2-AS1 on *SREBF2* transcription. TCGA LIHC dataset also showed that the expression of SREBF2 was positively correlated with FXR1 and TET1 expression in HCC tissues (Fig. 6N, O), supporting the SREBF2-AS1/FXR1/TET1/SREBF2 regulatory axis.

### SREBF2 mediates the oncogenic roles of SREBF2-AS1 in HCC

To investigate whether SREBF2 was the functional mediator of SREBF2-AS1 in HCC, we silenced SREBF2 expression in SREBF2-AS1 overexpressed HuH-7 cells (Supplementary Fig. S6A). CCK-8 and EdU incorporation assays showed that depletion of SREBF2 largely reversed the increased cell proliferation caused by SREBF2-AS1 overexpression (Supplementary Fig. S6B, C). TUNEL assays showed that depletion of SREBF2 reversed the decreased cell apoptosis caused by SREBF2-AS1 overexpression (Supplementary Fig. S6D). Transwell migration assays showed that depletion of SREBF2 largely reversed the increased cell migration caused by SREBF2-AS1 overexpression (Supplementary Fig. S6E). Cell viabilities measurement further showed that depletion of SREBF2 largely reversed the increased sorafenib resistance caused by SREBF2-AS1 overexpression (Supplementary Fig. S6F). These data suggested that SREBF2 at least partially mediated the oncogenic roles of SREBF2-AS1 in HCC.

## Discussion

Many reports, including our previous studies, identified several prognosis-correlated lncRNAs in HCC^44,45,56–58^. Aberrant m^6^A modification levels of lncRNAs were also found in HCC^21^. However, the correlation between m^6^A modification level of lncRNAs and prognosis in HCC is still largely unknown. In this study, we identified m^6^A-modified SREBF2-AS1 as a novel prognosis-related m^6^A modification event in HCC. We identified three m^6^A modification sites on SREBF2-AS1. Not only the expression level, but also the m^6^A modification level of SREBF2-AS1 was increased in HCC tissues and cells. Furthermore, not only the expression level, but also the m^6^A modification level of SREBF2-AS1 was correlated with poor prognosis of HCC patients. The m^6^A modification level of SREBF2-AS1 showed a higher hazard ratio (HR) than the expression level of SREBF2-AS1 in survival analyses. Thus, this study suggested m^6^A modification level of SREBF2-AS1 as a potential prognostic biomarker for HCC.

Functional investigations showed that SREBF2-AS1 exerted oncogenic roles in HCC, including promoting HCC cellular proliferation and migration, repressing HCC cellular apoptosis, and enhancing sorafenib resistance. The oncogenic roles of SREBF2-AS1 were dependent on m^6^A modification, as mutation of the m^6^A modification sites abolished the oncogenic roles of SREBF2-AS1 in HCC. Previous studies mainly found that m^6^A modification modulated the fate of RNAs, such as the processing, stability, and/or translation^20–22^. m^6^A modification exerted roles through changing the levels of RNAs or downstream products of RNAs ^59,60^. In this study, we demonstrated that m^6^A modification directly influenced the function of target RNAs.

Mechanistic investigations identified SREBF2 as the critical downstream target of SREBF2-AS1. The upregulation of SREBF2 by SREBF2-AS1 was also dependent on m^6^A modification of SREBF2-AS1. Our findings showed that although both m^6^A-modified and non-modified SREBF2-AS1 bound to *SREBF2* promoter, only m^6^A-modified SREBF2-AS1 regulated the transcription of *SREBF2*. Consistent with previous report about the roles of RNA m^6^A modification in regulating transcription via DNA demethylation^55^, here we showed m^6^A-modified SREBF2-AS1 as a concrete example. m^6^A-modified SREBF2-AS1 bound to the m^6^A reader FXR1, which further bound DNA 5-methylcytosine dioxygenase TET1. Thus, m^6^A-modified SREBF2-AS1 bound and recruited FXR1 and TET1 to *SREBF2* promoter, leading to DNA demethylation at *SREBF2* promoter and transcriptional activation of *SREBF2*. Non m^6^A-modified SREBF2-AS1 did not bind to FXR1, and therefore did not regulate DNA methylation of *SREBF2* promoter although non-modified SREBF2-AS1 also bound to *SREBF2* promoter. Functional rescue assays showed that depletion of SREBF2 largely reversed the oncogenic roles of SREBF2-AS1 in HCC.

**Figure 7:**
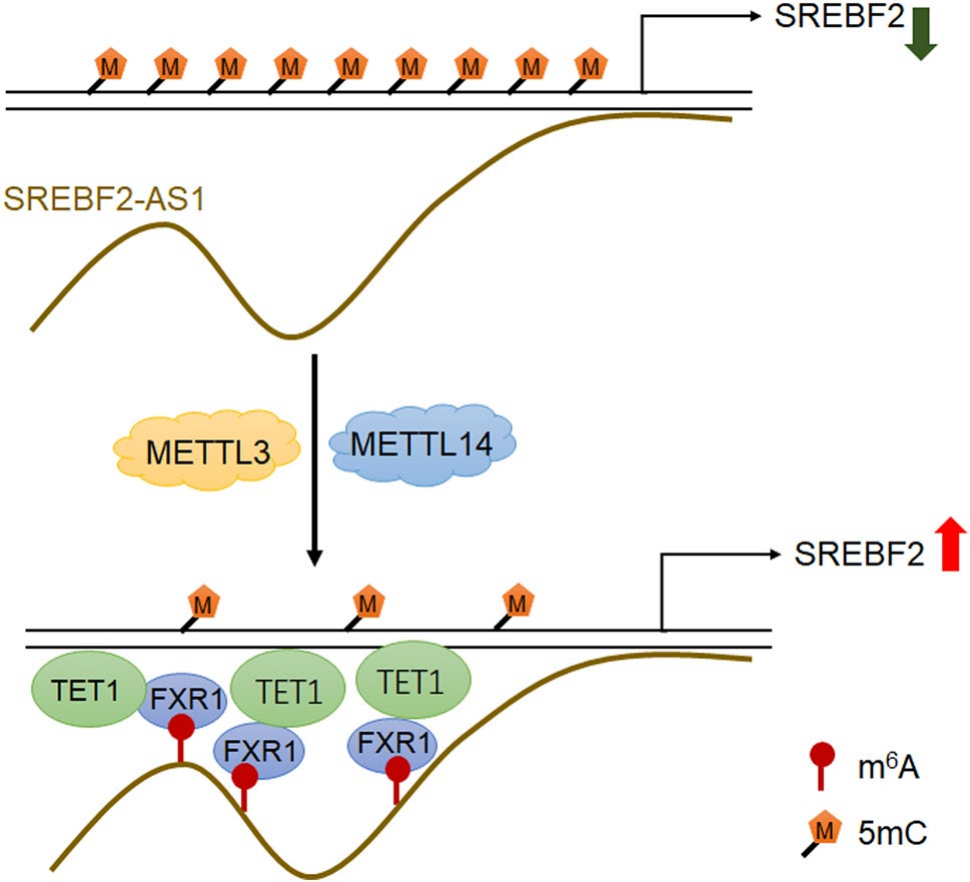
Schematic of the modulatory mechanisms of m^6^A-modified SREBF2-AS1 on DNA demethylation of *SREBF2*.

In summary, we identified a novel m^6^A modification event, which is m^6^A-modified SREBF2-AS1. m^6^A modification level of SREBF2-AS1 is increased in HCC and correlated with poor overall survival of HCC patients. m^6^A methyltransferases METTL3 and METTL14 induces m^6^A modification of SREBF2-AS1. m^6^A-modified SREBF2-AS1 binds to the m^6^A reader FXR1 and further DNA 5-methylcytosine dioxygenase TET1. Furthermore, SREBF2-AS1 binds to *SREBF2* promoter. Thus, m^6^A-modified SREBF2-AS1 binds and recruites FXR1 and TET1 to *SREBF2* promoter, leading to DNA demethylation at *SREBF2* promoter and transcriptional activation of *SREBF2* (Fig. 7). Through inducing SREBF2 upregulation, m^6^A-modified SREBF2-AS1 promotes HCC progression and sorafenib resistance. This study suggested m^6^A-modified SREBF2-AS1 as a prognostic biomarker and therapeutic target for HCC.

### Supplementary Information

Supplementary Legends.

Supplementary Figure 1.

Supplementary Figure 2.

Supplementary Figure 3.

Supplementary Figure 4.

Supplementary Figure 5.

Supplementary Figure 6.

Supplementary Figure 7.

Supplementary Figure 8.

## Data Availability

The TCGA LIHC datasets analyzed during the current study were download from https://portal.gdc.cancer.gov/. Other datasets generated and/or analyzed during the current study are available from the corresponding author on reasonable request.
