# Supplementary material for: METTL3 and METTL14-mediated N6-methyladenosine modification of SREBF2-AS1 facilitates hepatocellular carcinoma progression and sorafenib resistance through DNA demethylation of SREBF2
Source: Sci Rep. 2024 Mar 14;14:6155. doi: 10.1038/s41598-024-55932-7 (PMC10940719; doi:10.1038/s41598-024-55932-7)
Supplement: Supplementary file 1 — Supplementary Legends. [file 41598_2024_55932_MOESM1_ESM.docx]

**Supplementary figure legends**

**Supplementary Fig. S1** m^6^A modification level of SREBF2-AS1 in HCC cells. **A** m^6^A-modified SREBF2-AS1 was detected in HepG2 and HuH-7 cells using MeRIP assays with another primer pair for SREBF2-AS1. **B** m^6^A modification level of SREBF2-AS1 in immortalized liver cell line THLE-2 and HCC cell lines HepG2, HuH-7, and SNU-398 was measured by MeRIP assays with another primer pair for SREBF2-AS1. Results are shown as mean ± SD of 3 independent experiments. ***p* < 0.01, ****p* < 0.001, *****p* < 0.0001 by Student’s *t*-test (**A**) or one-way ANOVA followed by Dunnett's multiple comparisons test (**B**).

**Supplementary Fig. S2** METTL3 and METTL14-mediated m^6^A modification upregulates SREBF2-AS1 expression. **A** The correlation between SREBF2-AS1 and METTL16 expression level in 371 HCC tissues, derived from TCGA LIHC dataset. r = 0.0002, *p* = 0.9973 by Spearman correlation analysis. **B** METTL3 overexpression efficacy was detected by western blot. **C** METTL14 overexpression efficacy was measured by western blot. **D** m^6^A modification level of SREBF2-AS1 in HuH-7 cells with METTL3 or METTL14 overexpression was measured by MeRIP assays with another primer pair for SREBF2-AS1. **E** SREBF2-AS1 expression in HuH-7 cells with METTL3 or METTL14 overexpression was measured by RT-qPCR with another primer pair for SREBF2-AS1. **F** METTL3 depletion efficacy was detected by western blot. **G** METTL14 depletion efficacy was measured by western blot. **H** m^6^A modification level of SREBF2-AS1 in HuH-7 cells with METTL3 or METTL14 depletion was measured by MeRIP assays with another primer pair for SREBF2-AS1. **I** SREBF2-AS1 expression in HuH-7 cells with METTL3 or METTL14 depletion was measured by RT-qPCR with another primer pair for SREBF2-AS1. For **D**, **E**, **H** and **I**, results are shown as mean ± SD of 3 independent experiments. **p* < 0.05, ***p* < 0.01 by one-way ANOVA followed by Dunnett's multiple comparisons test. Original scans of blots are shown in Supplementary Fig. S7.

**Supplementary Fig. S3** The mutation of three m^6^A modification sites on SREBF2-AS1 abolishes the m^6^A modification of SREBF2-AS1. **A**, **B** SREBF2-AS1 expression was measured by RT-qPCR with another primer pair for SREBF2-AS1 in HuH-7 (**A**) or HepG2 (**B**) cells with stable overexpression of wild-type or three m^6^A modification sites mutated SREBF2-AS1. **C**, **D** m^6^A modification level of mutated SREBF2-AS1 was detected in HuH-7 (**C**) or HepG2 (**D**) cells using MeRIP assays with primer pair for mutated SREBF2-AS1. Results are shown as mean ± SD of 3 independent experiments. ****p* < 0.001, *****p* < 0.0001, ns, not significant, by one-way ANOVA followed by Dunnett's multiple comparisons test (**A**) or Student’s *t*-test (**B**).

**Supplementary Fig. S4** Depletion of SREBF2-AS1 represses oncogenic properties and sorafenib resistance of HCC cells. **A** SREBF2-AS1 expression was measured by RT-qPCR in HuH-7 cells with stable depletion of SREBF2-AS1. **B** SREBF2-AS1 expression was measured by RT-qPCR with another primer pair for SREBF2-AS1 in HuH-7 cells with stable depletion of SREBF2-AS1. **C** Cellular proliferation of HuH-7 cells with depletion of SREBF2-AS1 was measured by CCK-8 assays. **D** Cellular proliferation of HuH-7 cells with depletion of SREBF2-AS1 was measured by EdU incorporation assays. Scale bars, 100 µm. **E** Cellular apoptosis of HuH-7 cells with depletion of SREBF2-AS1 was measured by TUNEL assays. Scale bars, 100 µm. **F** Cellular migration of HuH-7 cells with depletion of SREBF2-AS1 was measured by transwell migration assays. Scale bars, 100 µm. **G** Cell viability was measured by Glo cell viability assays in HuH-7 cells with depletion of SREBF2-AS1 after sorafenib treatment, normalized to no sorafenib treatment. Results are shown as mean ± SD of 3 independent experiments. **p* < 0.05, ***p* < 0.01, ****p* < 0.001 by one-way ANOVA followed by Dunnett's multiple comparisons test.

**Supplementary Fig. S5** SREBF2-AS1 upregulates SREBF2 expression. **A** SREBF2 expression was measured by western blot in HuH-7 cells with stable overexpression of wild-type or m^6^A modification sites mutated SREBF2-AS1. **B** SREBF2 expression was measured by western blot in HuH-7 cells with stable depletion of SREBF2-AS1. **C** Ectopic expression of SREBF2-AS1 rescued the expression of SREBF2 which was repressed by SREBF2-AS1 depletion. **D** Ectopic expression of SREBF2-AS1 rescued the expression of STARD4 which was repressed by SREBF2-AS1 depletion. Results are shown as mean ± SD of 3 independent experiments. **p* < 0.05, ***p* < 0.01, ns, not significant, by one-way ANOVA followed by Dunnett's multiple comparisons test. Original scans of blots are shown in Supplementary Fig. S8.

**Supplementary Fig. S6** Depletion of SREBF2 reverses the oncogenic roles of SREBF2-AS1 in HCC. **A** SREBF2-AS1 and SREBF2 expressions were measured by RT-qPCR in HuH-7 cells with SREBF2-AS1 overexpression and concurrent SREBF2 depletion. **B** Cellular proliferation of HuH-7 cells with SREBF2-AS1 overexpression and concurrent SREBF2 depletion was measured by CCK-8 assays. **C** Cellular proliferation of HuH-7 cells with SREBF2-AS1 overexpression and concurrent SREBF2 depletion was measured by EdU incorporation assays. Scale bars, 100 µm. **D** Cellular apoptosis of HuH-7 cells with SREBF2-AS1 overexpression and concurrent SREBF2 depletion was measured by TUNEL assays. Scale bars, 100 µm. **E** Cellular migration of HuH-7 cells with SREBF2-AS1 overexpression and concurrent SREBF2 depletion was measured by transwell migration assays. Scale bars, 100 µm. **F** Cell viability was measured by Glo cell viability assays in HuH-7 cells with SREBF2-AS1 overexpression and concurrent SREBF2 depletion after sorafenib treatment. Results are shown as mean ± SD of 3 independent experiments. **p* < 0.05, ***p* < 0.01, ****p* < 0.001, *****p* < 0.0001, ns, not significant, by one-way ANOVA followed by Dunnett's multiple comparisons test.

**Supplementary Fig. S7** Whole original western blot images for Supplementary Figure S2.

**Supplementary Fig. S8** Whole original western blot images for Supplementary Figure S5.
