# Supplementary figures and images for: METTL3 and METTL14-mediated N6-methyladenosine modification of SREBF2-AS1 facilitates hepatocellular carcinoma progression and sorafenib resistance through DNA demethylation of SREBF2
Source: Sci Rep. 2024 Mar 14;14:6155. doi: 10.1038/s41598-024-55932-7 (PMC10940719; doi:10.1038/s41598-024-55932-7)

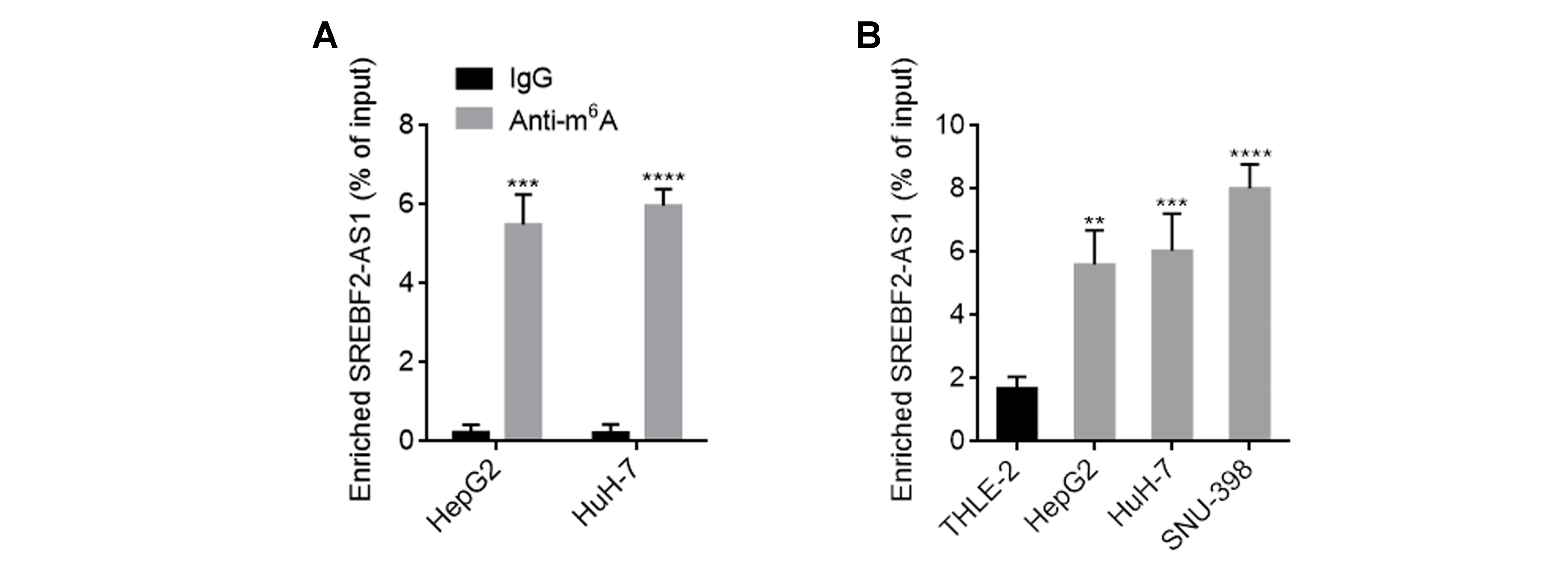

Supplement: Supplementary file 2 — Supplementary Figure 1. [file 41598_2024_55932_MOESM2_ESM.tif]

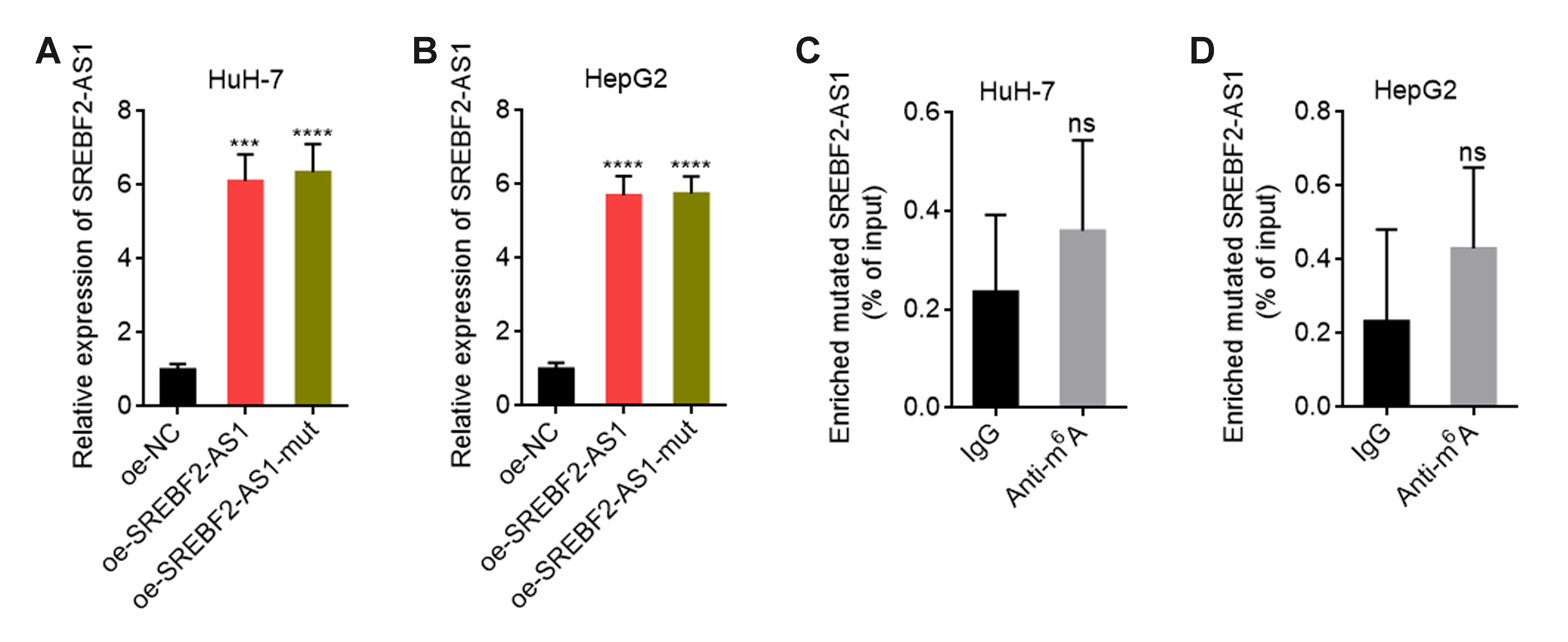

Supplement: Supplementary file 4 — Supplementary Figure 3. [file 41598_2024_55932_MOESM4_ESM.tif]

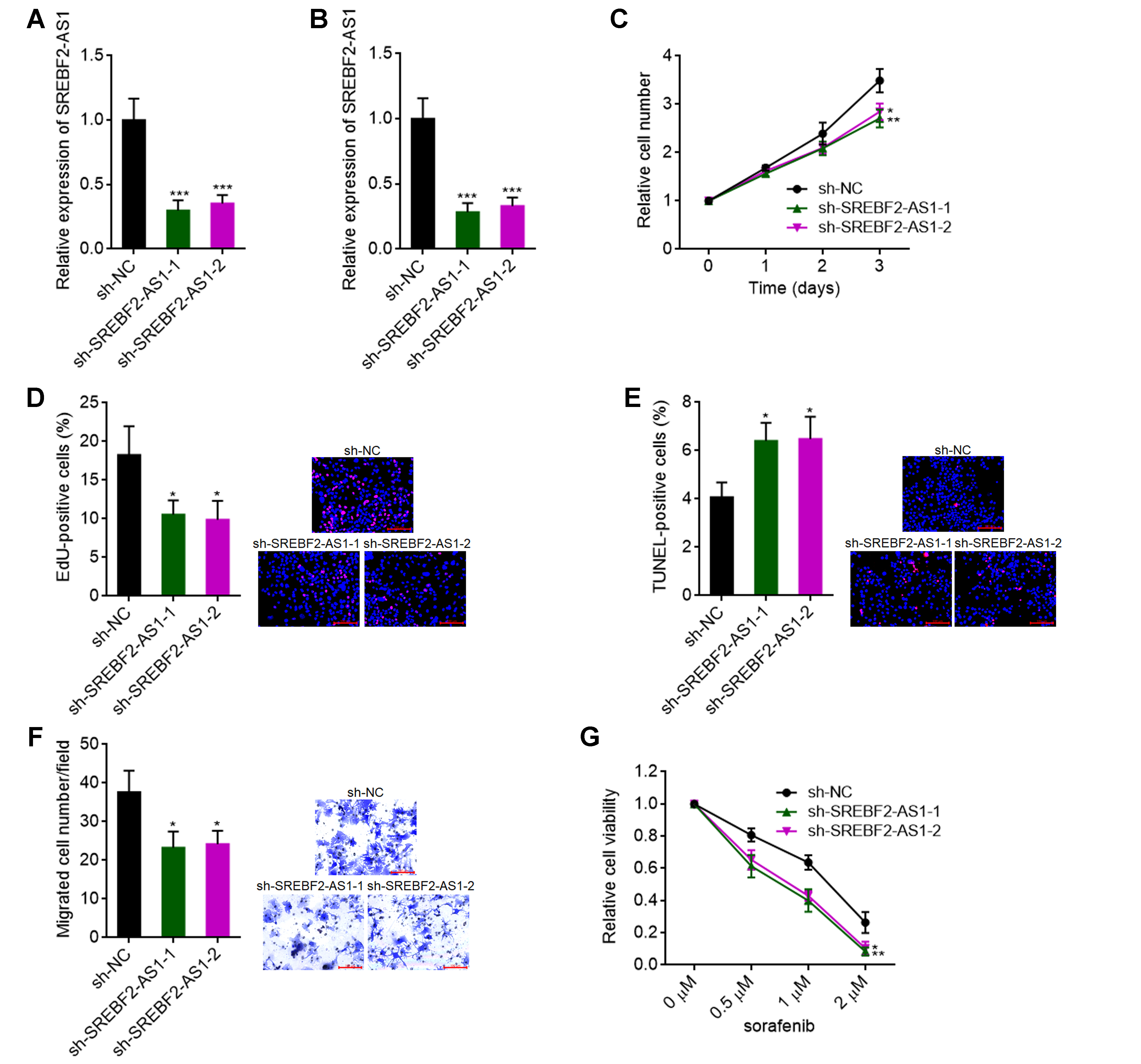

Supplement: Supplementary file 5 — Supplementary Figure 4. [file 41598_2024_55932_MOESM5_ESM.tif]

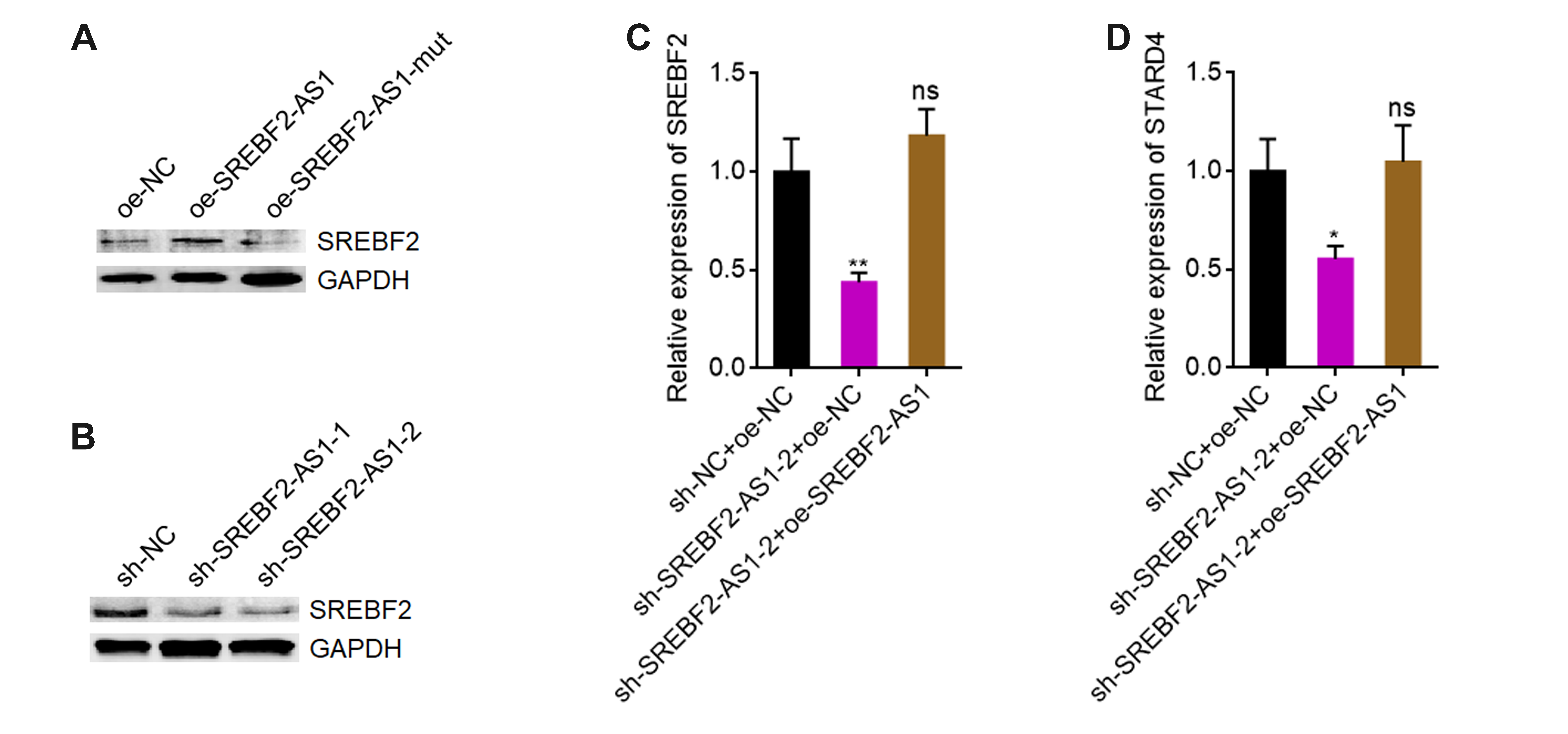

Supplement: Supplementary file 6 — Supplementary Figure 5. [file 41598_2024_55932_MOESM6_ESM.tif]

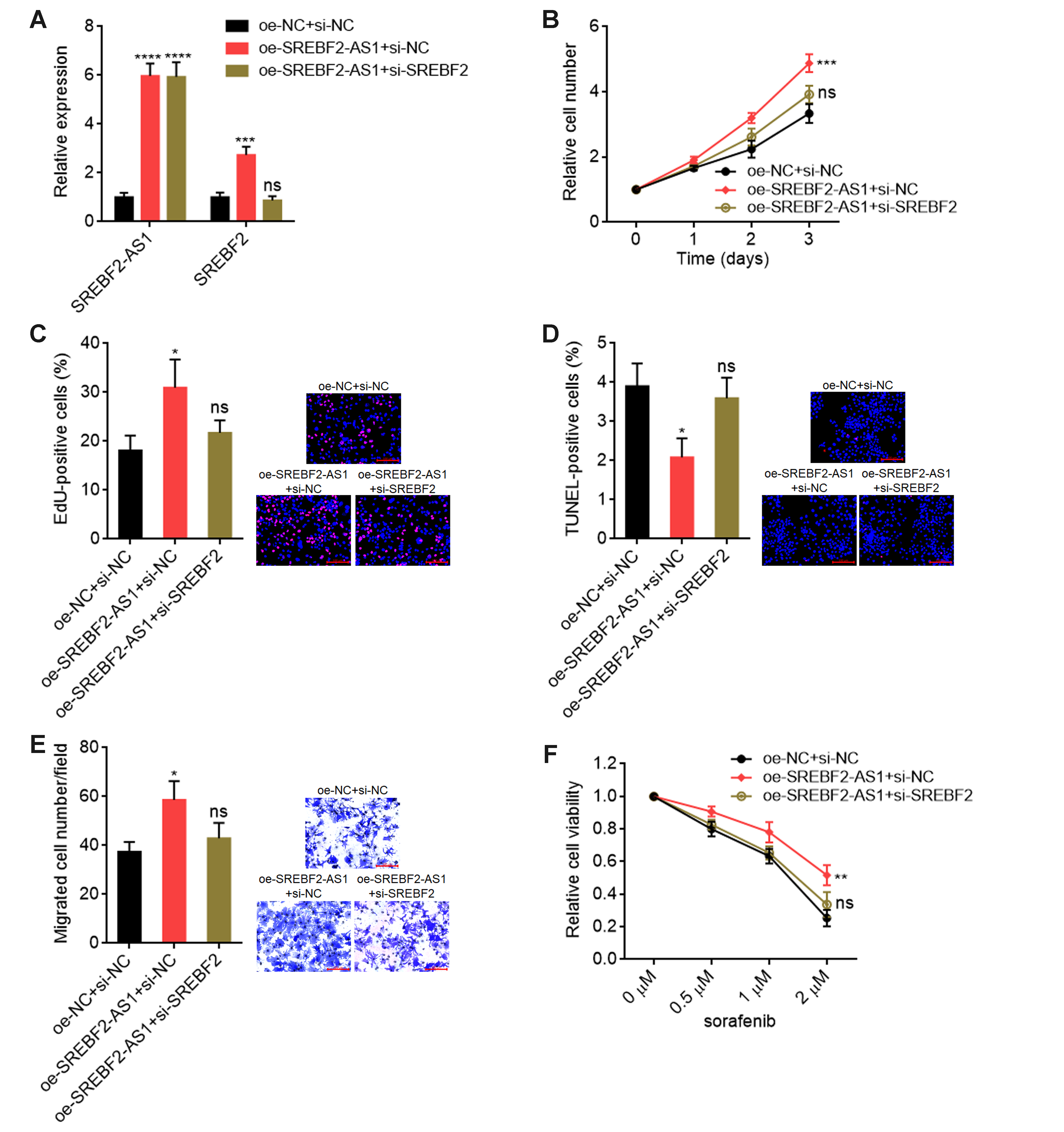

Supplement: Supplementary file 7 — Supplementary Figure 6. [file 41598_2024_55932_MOESM7_ESM.tif]

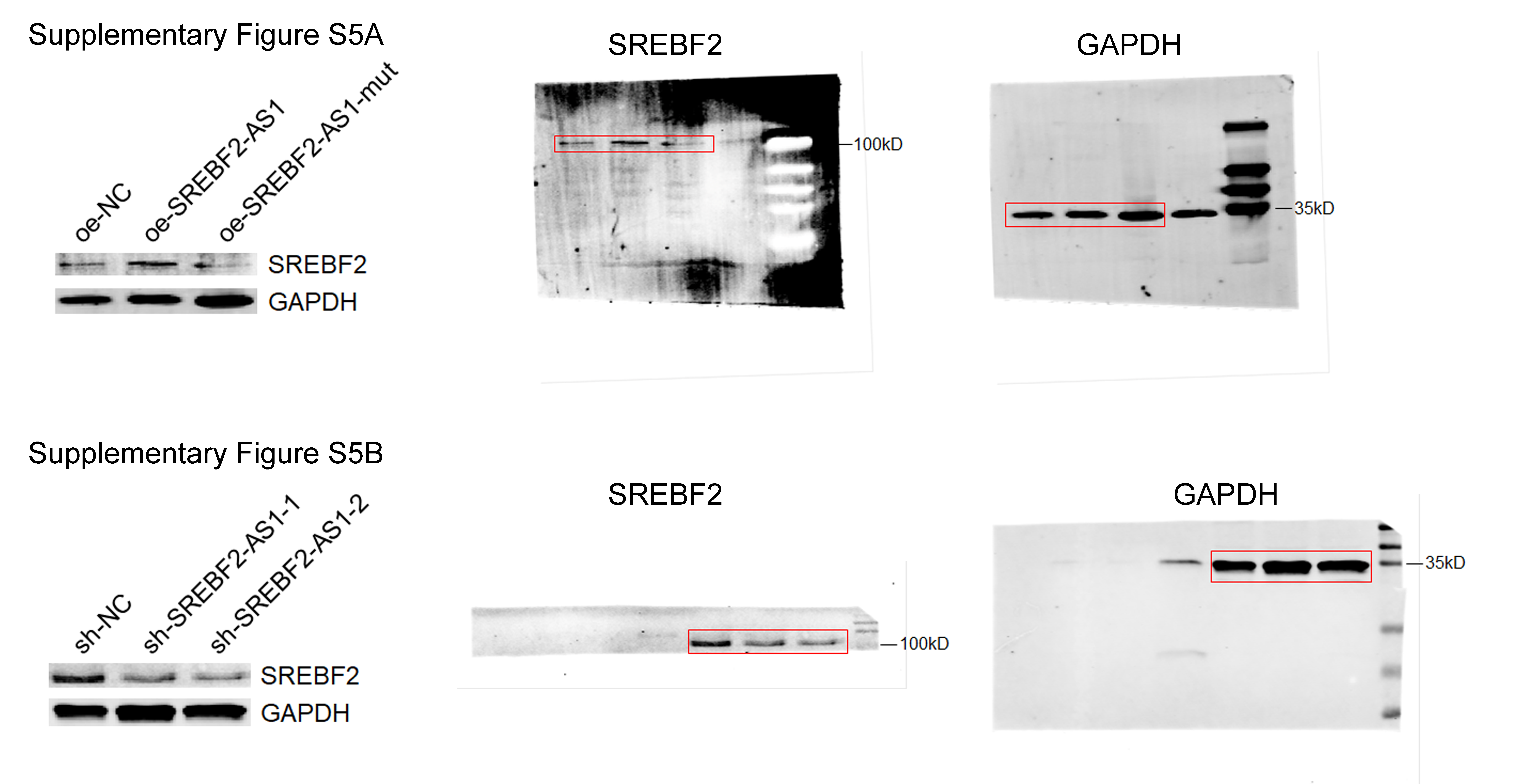

Supplement: Supplementary file 9 — Supplementary Figure 8. [file 41598_2024_55932_MOESM9_ESM.tif]
